# Methods for statistical fine-mapping and their applications to auto-immune diseases

**DOI:** 10.1007/s00281-021-00902-8

**Published:** 2022-01-18

**Authors:** Qingbo S. Wang, Hailiang Huang

**Affiliations:** 1 Department of Statistical Genetics, Osaka University Graduate School of Medicine, Osaka, Japan; 2 Department of Biomedical Informatics, Harvard Medical School, Boston, MA USA; 3 Analytic and Translational Genetics Unit, Massachusetts General Hospital, Boston, MA USA; 4 Program in Medical and Population Genetics, Broad Institute of MIT and Harvard, Cambridge, MA USA; 5 Stanley Center for Psychiatric Research, Broad Institute of MIT and Harvard, Cambridge, MA USA; 6 Department of Medicine, Harvard Medical School, Boston, MA USA

**Keywords:** Statistical fine-mapping, Functionally informed fine-mapping, Bayesian, Autoimmune disorders, Inflammatory bowel diseases, IBD genetics

## Abstract

Although genome-wide association studies (GWAS) have identified thousands of loci in the human genome that are associated with different traits, understanding the biological mechanisms underlying the association signals identified in GWAS remains challenging. Statistical fine-mapping is a method aiming to refine GWAS signals by evaluating which variant(s) are truly causal to the phenotype. Here, we review the types of statistical fine-mapping methods that have been widely used to date, with a focus on recently developed functionally informed fine-mapping (FIFM) methods that utilize functional annotations. We then systematically review the applications of statistical fine-mapping in autoimmune disease studies to highlight the value of statistical fine-mapping in biological contexts.

## Introduction

**Fig. 1:**
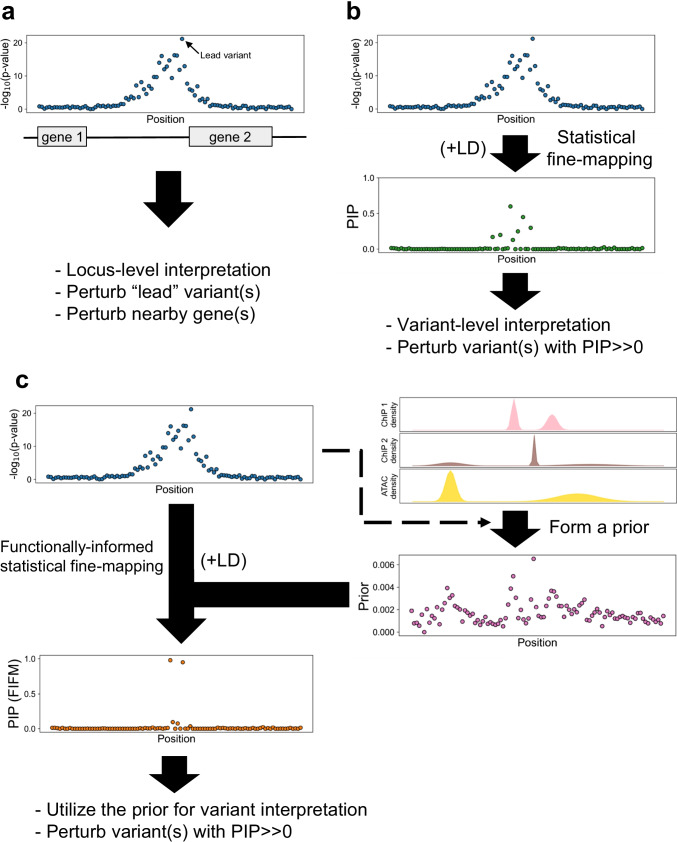
Schematic overview of the statistical fine-mapping methods with uniform or functionally informed prior, in comparison with direct experimental approaches. **a** Downstream experiments following GWAS without statistical fine-mapping often assume the variant with the most significant *p*-value (“lead” variant) as the causal variant and proceed to perturbation of the lead variant and/or nearby gene(s). **b** Statistical fine-mapping is utilized to prioritize a small number of variants for downstream perturbation, which can be different from what *p*-value in GWAS suggests. This facilitates variant-level interpretation of GWAS results. **c** In a functionally informed fine-mapping (FIFM) framework, functional annotations are used (often together with the GWAS data) to form a prior. FIFM often results in an increase of power in prioritizing putative causal variants, which is typically characterized by higher maximum posterior inclusion probability (PIP) and/or lower credible set size^14^. The functional annotations used to form the prior are often directly used to interpret the biological mechanisms of causal variant(s)

Genome-wide association studies (GWAS) have identified thousands of loci in the human genome that are associated with different traits such as height, body mass index (BMI), or susceptibility to different diseases [1–3]. In typical GWAS, for phenotype and genotype of interest, their relationship is modeled in a generalized linear model such that the phenotype (either quantitative or logit of binary outcome) is the sum of the genotype times its effect size (slope), the effects of covariates such as sex, age, and principal components accounting for the population structure, intercept, and error term [4, 5] (Box. 1). The null hypothesis that the slope is zero (i.e., the genotype of interest is not associated with the phenotype of interest) is tested for each variant where the genotype is available. In other words, each variant will have a *p*-value that characterizes the evidence that the variant is associated with the phenotype in a frequentist approach. With proper quality control and rigorous correction for multiple test, the variants passing significance threshold [6] (typically 5 × 10^−8^, so called “genome-wide significance”) is considered to be associated with the phenotype of interest. However, there is a clear difference between association and causation. This is true in GWAS as well: studies [7, 8] suggest that the majority of variants with significant *p*-value (i.e., “associated” with phenotypes) will have no detectable effect on the phenotype when perturbed (i.e., “causal” to phenotype). Such observations motivate us to differentiate between “association” and “causality,” to pinpoint the causal variant(s) in a locus. Fine-mapping [9, 10] is such an effort to pinpoint causal variants (either experimentally or computationally), and statistical fine-mapping [9, 11] is a subset of fine-mapping studies that utilizes statistical framework. In this review, we will discuss the nature of statistical fine-mapping with four focuses. First, we will briefly review the challenges of GWAS as well as experimental perturbation approaches to further clarify the motivation of statistical fine-mapping. Second, we will review the types of statistical fine-mapping methods that have been widely used to date (Fig. 1). Since high-quality reviews that achieve the same purpose already exist [9, 11, 12], we will make this second section brief without deep-diving into individual methods. On the other hand, a number of large-scale statistical fine-mapping studies [13, 14] have emerged recently. Since a large majority of such studies utilize functional annotations to perform functionally informed fine-mapping (FIFM), our third focus will be on the application of FIFM on large-scale studies. Finally, to highlight the value of statistical fine-mapping in biological contexts, we will systematically review the applications of statistical fine-mapping in autoimmune disease studies.

## GWAS is not designed for causal variant identification

**Fig. 2:**
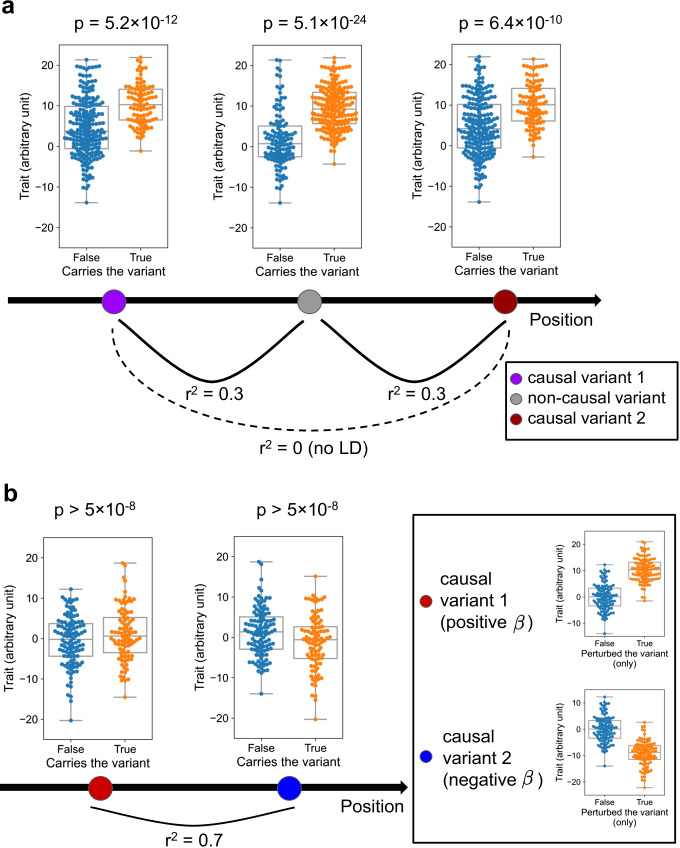
Two simplified examples where marginal *p*-value fails to prioritize the true causal variants. **a** The non-causal variant (center), frequently tagging one of the two true causal variants, has the most significant association *p*-value (in *F*-test) as well as the highest marginal effect size $\beta$ (7.8 vs 5.8 and 5.3). **b** Two nearby causal variants in LD harboring high true effect sizes to the opposite direction, both have limited marginal association *p*-values that do not reach the statistical significance under multiple test correction (*p* = 0.06 and 0.007). Synthetic samples of *n* = 300 for **a** and *n* = 200 for **b** were generated, with true $|\beta |$ = 10 and $\epsilon$ drawn from a normal distribution with SD = 5 for simplicity (therefore, *y* axis has no unit). *r* = 0.317, 0.317, and 0.0353 for **a** and 0.734 for **b** The code is available at http://github.com/QingboWang/fm-toy

GWAS is not designed for identifying causal variants at single-variant resolution — instead, GWAS is designed to identify regions in the genome that are associated with the trait of interest [15, 16]. The main factor that makes association and causality different in GWAS is the existence of linkage disequilibrium (LD). LD is a term that describes the non-random association between nearby genomic variants [17, 18] — variants that are nearby tend to appear together more (or less) often than by chance, because the probability that a recombination event occurs at a position between two variants are typically smaller when they are nearby, compared to when they are far away. The strength of LD between two variants is typically denoted by the Pearson correlation coefficient (*r*) [18]. Because of LD, even if there is only one causal variant in a locus, hundreds or thousands of non-causal variants can be associated with the phenotype in GWAS, just because they are associated with the causal variant [19, 20] (i.e., “tagged”). In fact, the set of variants tested for association in GWAS, either directly measured in a genotyping array [21, 22] or imputed from a population reference panel [15, 23], are typically common variants that are designed to hopefully “tag” the causal variant. They are, most of the time, no more than “markers” that are in LD with causal variant(s), and the causal variant(s) themselves can even be missing from the set of variants tested. In addition, due to the limited sample size and stochastic noises, the variant with the strongest association (i.e., “lead variant,” the variant with lowest *p*-value) is not always the causal variant. Understanding which variant(s) are truly causal (in other words, identifying the true causal configuration) can be even more complicated since there are often more than one causal variant in a locus (a locus is typically defined as a set of variants with (*r*^2^ against the lead variant) > threshold, or simply within a certain distance window [24]). In such cases, the effect size and direction we observe for a variant can largely vary from the true causal effect (Fig. 2). Statistical fine-mapping, in a way, can be thought of as an exercise to disentangle the effect of LD from the GWAS data.

These factors remind us of the fact that even with orders of magnitude larger sample sizes, GWAS alone is, by nature, still not suited for causal variant identification, and highlight the value of statistical fine-mapping methods.

## Experimental approaches are valuable but limited

Since one of the goals of GWAS is to nominate a set of regions for downstream biological experiments, one natural suggestion would be to directly move to experimental validations after GWAS, without performing statistical fine-mapping. One caveat of such approaches is that it often ambiguates the biological mechanisms underlying the GWAS signal. As a toy example, if a locus of gene X is associated with phenotype Y, we can validate that X is causal for Y by knocking out the entire gene X. However, if we can statistically fine-map the causal variant V on gene X and validate it by introducing variant V at single base-pair resolution followed by different biological assays, we can highlight different scenarios such as V introducing a stop codon, V being a missense variant that changes the protein 3D conformation, and V introducing aberrant splicing (Fig. 1a,b).

Recent developments in such genome perturbation at single-variant resolution have been remarkable. (1) Massive parallel reporter assay [7, 8, 25, 26] enables us to test the effect of mutations on gene expression in vitro with high throughput. (2) Genome engineering tools such as base editors enable introduction of single base-pair mutations in vivo [27–29]. However, they are still limited in that (1) is not a perfect proxy of human physiology and (2) is limited in its throughput, such that saturation mutagenesis at genome-wide scale is still far away. Performing statistical fine-mapping before such experimental validation is thus a natural way to maximize the value of downstream experimental approaches. Developments in the methods so called co-localization [30–34] further enhanced the value of statistical fine-mapping, by analyzing the results of statistical fine-mapping on complex traits and gene expression regulation (expression quantitative loci, or eQTL) studies simultaneously to elucidate the mechanisms from variant to gene to complex trait in a streamlined manner, making the downstream experimental validation easily designable and interpretable.

## From *p*-value to Bayes factor and Posterior inclusion probability

Although *p*-value characterizes the evidence of a variant being associated with the phenotype, it does not allow us to compare one model likelihood (e.g., a model that variant V1 is causal) to another (e.g., a model that variant V2 is causal) in a direct and quantitative way. Bayes factor (BF) is a notion that quantifies the relative likelihood of one model over another [35] (Box. 2). Early studies [23, 36] included such a Bayesian approach and reported BF in addition to canonical *p*-value, with a hint to use it as a means to directly quantify the “probability of being a causal variant.” Wakefield (2007, 2009) [37, 38] later showed that BF can be approximated from summary statistics (such as *p*-value, point estimation, and standard error of the effect size of each variant from GWAS) without individual-level genotype data (“Approximate Bayes Factor,” or “ABF”). With these developments in Bayesian approaches to GWAS, Maller et al. [39] showed that, under a simplified scenario that there is exactly one causal variant in a locus of interest, we can directly compute the probability of a variant V being causal (nowadays called posterior inclusion probability, or PIP), as the BF of the model that V is causal (compared to the model that no variant is causal; they showed that this BF can be calculated by the genotype of variant V alone), divided by the sum of BF that each of the other variants is causal. They also introduced the term credible set, defined as the smallest set of variants that the PIPs sum up to a certain threshold value.

Statistical fine-mapping assuming a single causal variant in a locus has been valuable in its simplicity and interpretability, but it relies on a very strong assumption that does not necessarily hold true. Maller et al. [39], fully aware of the fact, also suggested that jointly modeling multiple causal variants is theoretically possible, but the implementation is challenging (when there are *n* variants, there would be 2^*n*^ causal configurations). One of the main focuses in the later development of statistical fine-mapping methods lays on modeling multiple causal variants. We also note that, although Bayesian approaches force us to specify the prior by nature (which could introduce biases) and non-Bayesian statistical fine-mapping approaches exist [40], our methods review will be focused on Bayesian approaches that are most commonly used.

## Multiple causal variants

One possible approach for dealing with locus that may harbor more than one causal variant is to divide up the set of variants into independent signals, such that each set of variants would contain exactly one causal variant. Although this intuitively could be achieved by a series of conditional analysis (i.e., condition on the lead variant by including it as a covariate, do GWAS again to find remaining signal, add the lead variant in that conditioned GWAS, do the GWAS again, … until we see no more signal), it introduces practical challenges; setting the *p*-value threshold to determine that there is no more signal is non-trivial, and running GWAS iteratively is computationally expensive [11]. An early study [41] avoided such challenges and practiced a simple approach to define a locus simply based on the *r*^2^ against the lead variant (i.e., clumping and merging) and to assume that each locus contains exactly one causal variant. Yang et al. [42] showed that without such extreme model simplification, conditioning can be achieved with summary statistics and LD matrix without requiring individual genotype data in a scalable manner. Once a set of variants likely containing exactly one causal variant is defined, ABF can be applied also from summary statistics. Such a COJO + ABF approach allows the whole process of identifying multiple causal variants tractable, by dividing up the problem into two steps: (1) identify a set of variants harboring exactly one causal variant, and (2) perform ABF for each variant set. The COJO + ABF approach has been widely used since then [43, 44].

However, over the time, there has been increasing amounts of evidence that conditional analysis often results in sub-optimal solutions, in simulations [11, 45–47] and real data [48, 49]. The simplest intuition [45] is that a non-causal variant that is in strong LD with two causal variants can have the most significant *p*-value and thus be mistakenly prioritized as the causal variant (Fig. 2a). One of the major methods that overcomes this limitation was presented in Hormozdiari et al. [45] (CAVIAR). In CAVIAR, they took the approach of jointly modeling multiple causal variants rather than sequentially, and allowed directly calculating the BF for the case of > 1 causal variant. They dealt with the computational complexity by limiting the maximum number of causal variants as well as the number of variants in the locus. Other approaches that are distinct from naive conditional approach includes [BIMBAM [50]] (requires individual genotype data), [pi-MASS [51]] (utilizes MCMC), [JAM [47]] (utilizes matrix decomposition), the extensions of CAVIAR that is more scalable and widely applicable [CAVIARBF [52], eCAVIAR [31]], and those used in autoimmune disease studies that are discussed later [53].

## Scalable methods

Although methods such as CAVIAR allowed a joint model of multiple causal variants, scaling such methods to a genome-wide level remained challenging. To implement a scalable fine-mapping method, Benner et al. [54] applied a shotgun stochastic search of the possible causal configurations instead of exhaustively enumerating the BFs for 2^*n*^ causal configurations. Their method, FINEMAP, either adds, exchanges, or deletes one putative causal variant in the locus in each iteration to generate a new causal configuration to evaluate. The method further utilizes a hash table to avoid re-computation of the same causal configuration and terminates the iteration once nearly all the causal configurations with non-negligible probability are searched. Wen et al. [55] (DAP-G) used a similar idea of avoiding the enumeration of all 2^*n*^ causal configurations by focusing only on non-negligible ones but used a deterministic method instead; their Deterministic Approximation of Posterior (DAP) algorithm restricts the search space based on two assumptions: (1) The true causal variants should have medium to highly significant association *p*-value. (2) The fraction of causal variants in a locus should be small (sparsity assumption) and allows tractable computation. Another widely used method developed by Wang et al. [56] (Sum of Single Effects = SuSiE) takes an iterative approach; analogous to conditional analysis, SuSiE takes single effect regression (a regression model where there is exactly one causal variant in a locus) as a building block to perform iterative Bayesian stepwise selection (IBSS). The algorithm (1) explicitly specifies L single effect vectors (initialized with uniform probability of being causal for each variant in each single effect vector, when the prior is uniform) to begin with, updates the 1st single effect vector based on the data, and (2) repeats the process of updating the 2nd, 3rd, …, L-th, 1st, 2nd, … single effect vector based on the data plus all the other single effect vectors until convergence.

Each of these methods is highly scalable and has been applicable to different large-scale studies (e.g., DAP-G in GTEx v8 study [57] and FINEMAP for UKBB biomarkers study in Sinnott-Armstrong et al. [58]) to elucidate the detailed biological mechanisms of GWAS signals, highlighting the value of scalable methods.

## Functionally informed fine-mapping

A variant falling in a histone mark peak is more (or less) likely to be causal to a phenotype compared to another variant. A missense variant is more likely to be causal than an intron variant. Such additional biological information about the variants (e.g., epigenetic information, conservation, or other scores, which are called “functional annotations”) are informative to identify causal variants, even before investigating specific GWAS data. In other words, we have a “prior” knowledge about the variants. As one strength of Bayesian methods is that it can flexibly incorporate different priors, a number of methods including those highlighted in the previous section  [55, 59–63] allow incorporating such biological functional annotations as priors to increase the power of statistical fine-mapping. For example, distance to transcription starting site (dTSS) was incorporated as a prior in DAP-G to perform *cis*-eQTL fine-mapping in GTEx v8 [57]. We call such a series of methods that use functional annotations to form a prior (rather than using functional annotations post-statistical fine-mapping only to interpret the results; Fig. 1c) as functionally informed (statistical) fine-mapping (FIFM). Among various FIFM methods, this review focuses on two recent large-scale FIFM methods: (1) Polyfun [13] that was applied to UKBB phenotypes and (2) EMS [14] that was applied to GTEx v8 eQTLs. These two methods, rather than performing expectation–maximization (EM) iteration in the fine-mapping process as in PAINTOR [59], take a two-step approach of first calibrating the functional prior and then using the functional prior to perform FIFM using scalable methods [FINEMAP [54], SuSiE [56]].

The first method, Polyfun, allows the incorporation of functional features by stratified ld-score regression (S-LDSC [20]). First, it uses S-LDSC to estimate the heritability enrichment of each of the functional annotations for a phenotype of interest (with proper regularization and training-test split to avoid overfitting). Second, it estimates the per-SNP heritability (heritability explained by a single nucleotide polymorphism = SNP) by adding up the heritability enrichment of the functional annotations that the variant (SNP) of interest belongs to. Following the calibration step (binning the SNPs and re-calculating the per-SNP heritability for each bin), the functional prior is defined to be proportional to the per-SNP heritability. Then they use the functional prior for downstream statistical fine-mapping using SuSiE or FINEMAP. By applying the method to 49 UKBB traits, the authors validated the power gain of FIFM compared to canonical methods and also discussed the polygenic localization of common trait heritability (i.e., how many variants are needed to explain a certain percentage of trait heritability).

The second method, Expression Modifier Score (EMS), first trains a random forest (RF)-based predictor that uses > 6,000 functional annotations, to prioritize putative causal eQTLs that are nominated with high confidence in uniform prior fine-mapping. The method also includes deep-neural network-based variant activity prediction scores [64, 65] as a set of features and shows that those features collectively present high feature importance in addition to dTSS. In the subsequent step, the output scores (EMS) from the RF model are scaled and used to re-weight the single effect vectors in SuSiE. Functionally informed PIP and credible sets are then quantified from the weighted vectors for 49 GTEx tissues individually. The method was also applied for a large-scale co-localization analysis to elucidate > 300 additional candidate genes for UKBB phenotypes.

These results both showed an improvement compared to the canonical methods in terms of the number of putative causal variants discovered, without loss of accuracy, and thus together highlighted the value of performing FIFM on a large scale.

## Further extension of statistical fine-mapping methods

Although not deeply covered in this review, a more diverse set of applications exist in recent development of statistical fine-mapping methods [66–75]. First is the cross-population fine-mapping (xpop-FM) approach that utilizes different LD structures between populations. Such approaches [71, 72] rely on an assumption (supported by biological observations) that the true causal variant is, most of the time, shared between different populations [76, 77]. By simple intuition, when variant V0 and V1 each has PIP = 0.5 in population 1, variant V0 and V2 has PIP = 0.5 in population 2 and variant V0 and V3 has PIP = 0.5 in population 3, one would gain confidence that variant V0 is the true causal variant. One challenge in such xpop methods is the model misspecification, i.e., a causal variant may not be shared or has very different effects across populations for some loci (e.g., the *TNFSF15* locus, with Crohn’s disease OR of 1.15 and 1.75 for Europeans and East Asians respectively [78]). While such heterogeneity across populations can be properly modeled for GWAS using methods such as MANTRA [79], MR-MEGA [80], MAMA [81], or random effect models [82], the ability of xpop fine-mapping methods to model such heterogeneity has not been fully evaluated in real data. With further methodology developments as well as the increase in population diversity of the available genome, we envision the value of such xpop methods will increase. Similarly, harmonizing heterogeneous datasets with different underlying technologies (such as different arrays, whole exome, or genome sequencing) and including low-frequency variants is thought to be fruitful for further discovery of putative causal variants underlying human complex disorders by increasing the statistical power and the coverage of the genome. Another direction is the optimization of the prior distribution of the causal effect sizes [83, 84] (not the causal configuration); for example, Walters et al. [83] suggested Laplace prior could increase the statistical power compared to the commonly used normal distribution. As optimizing the prior is a non-trivial problem in Bayesian analysis in general, it could be also valuable to discuss the possibility of moving outside of the Bayesian world to practice statistical fine-mapping in a frequentist approach. As a general note, no single method for statistical fine-mapping today serves as a “gold standard,” and different methods rely on different assumptions. Interpreting the results from multiple different aspects, as will be discussed in the next sections, is of high importance.

## Application of statistical fine-mapping in autoimmune diseases

Many autoimmune disorders are highly heritable [85]. GWAS and statistical fine-mapping have thus been very effective in finding genetic variants underlying these disorders. Here, we review methods and findings for ten major autoimmune disorders including rheumatoid arthritis (RA), type 1 diabetes (T1D), the inflammatory bowel diseases (IBD) including Crohn’s disease (CD) and ulcerative colitis (UC), systemic lupus erythematosus (SLE), ankylosing spondylitis (AS), psoriasis (PSOR), autoimmune thyroid disease (THY), celiac disease (CeD), and multiple sclerosis (MS). We chose these disorders because they are sufficiently powered with at least 10,000 cases. The number of genetic loci associated with these disorders ranges from 40 (CeD) to 240 (IBD) and is influenced by the sample size, the heritability, and the genetic architecture of the disorder (Table 1).

**Table 1:** GWAS and fine-mapping analyses across ten autoimmune disorders. All studies were performed on European subjects except for SLE, which combined European and East Asian subjects in the fine-mapping analysis

Disorder	Abbreviation	Heritability (CIs) (c)	GWAS			Fine-mapping					
			# case	# loci	PMID	# case	# loci	1-SNP set	5-SNP set	Method	PMID
Ankylosing spondylitis	AS	0.97 (0.92–0.99)	10,417	48	26974007	10,619	28	0	-	PICS	25363779
Autoimmune thyroid disease	THY	0.79	30,234	93	32581359	2,747	10	0	-	PICS	25363779
Celiac disease	CeD	0.57 (0.32–0.93)	12,041	40	22057235	12,041	40	1	-	PICS	25363779
Inflammatory bowel diseases—Crohn’s disease (a)	IBD—CD	1.00 (0.34–1.00)	25,042	240	28067908	20,155	94	18	42	-	28658209
Inflammatory bowel diseases—Ulcerative colitis (a)	IBD—UC	0.67 ± 0.13				15,191					
Multiple sclerosis	MS	0.25 (0.00–0.88)	47,429	233	31604244	14,498	87	2	-	PICS	25363779
Psoriasis	PSOR	0.66 (0.52–0.77)	19,032	63	28537254	10,588	36	7	-	PICS	25363779
Rheumatoid arthritis	RA	0.68 (0.55–0.79)	22,628	121	33310728	11,475	46	0	5	ABF	30224649
Systemic lupus erythematosus	SLE	0.66	11,283	132	33536424	11,283	132	5	17	PAINTOR	33536424
Type 1 diabetes (b)	T1D	0.88 (0.78–0.94)	11,644	51	25751624	9,334	49	1	10	ABF	30224649

^a^CD and UC are two subtypes of IBD and are often analyzed together for their extensively shared genetic architecture

^b^The GWAS study included both case–control and family samples

^c^Heritability estimates compiled from multiple sources with detailed provided in Maria Gutierrez-Arcelus et al. Nature Reviews Genetics 2016 (PMID: 26907721)

Farh et al. [53] performed the first genome-wide statistical fine-mapping on several autoimmune disorders using Probabilistic Identification of Causal SNPs (PICS), an algorithm estimating the probability that an individual variant is causal considering the haplotype structure and observed pattern of association at the genetic locus. This fine-mapping analysis was performed on data available prior to July 2013. For some disorders (AS, PSOR, THY, CeD, and MS), this study remains the best available fine-mapping study. For other disorders (RA, T1D, CD, UC, and SLE), subsequent fine-mapping studies have been performed on data with larger sample size and higher quality (e.g., higher imputation quality and higher genomic coverage). These studies also used more sophisticated fine-mapping methods. RA and T1D used conditional analysis for multiple independent associations, and ABF to compute the credible sets. IBD used three fine-mapping methods specifically designed in order to capture the disease subtypes (CD and UC). Both the stepwise conditional analysis and MCMC were used to infer the independent associations for IBD. Results from the three methods were then harmonized which served as a quality control filter. SLE used conditional analysis for loci hosting multiple independent associations and PAINTOR [59] to compute the credible sets combining subjects of both European and East Asian ancestries. All fine-mapping studies for these disorders were performed without the functional priors.

Outcome of fine-mapping studies is dependent on the disease heritability, the sample size, and the disease genetic architecture (Tables 1 and 2). THY, MS, T1D, RA, and PSOR only mapped a subset of the genome-wide significant loci using a subset of subjects because the largest GWASs were published after the fine-mapping studies. None of the THY loci was mapped to a small credible set, likely because only less than 3,000 cases were used in fine-mapping. MS had two loci mapped to single-variant resolution, located in the introns of *RNASEL* and *HACE1*. T1D had one locus mapped to a single-variant credible set (*TYK2* P1104A) and nine more to credible sets with five or fewer variants. Fine-mapping for RA and PSOR was more productive: RA had five loci mapped to credible sets with five or fewer variants, and PSOR had seven loci mapped to a single causal variant, including the *TYK2* P1104A (also the T1D putative causal variant), a missense variant for *TRAF3IP2-AS1* (D10N), and variants in the introns of *KCNH7*, *DDX58*, and *NOS2*. AS, CeD, and SLE used all available GWAS samples. None of the AS loci was mapped to a small credible set likely because the effect sizes for AS loci are small thus are less powered for fine-mapping. One locus for CeD was mapped to a single variant (in the intron of *UBASH3A*), and 17 SLE loci were mapped to credible sets with five or fewer variants, among which five loci were mapped to a single causal variant, including a variant upstream of *TNFSF4* and a *WDFY4* missense variant (R1816Q). Driven by the sample size and the heritability, IBD fine-mapping is the most productive among the ten autoimmune disorders: 42 associations were mapped to credible sets with five or fewer variants, and 18 to a single causal variant, including multiple missense variants (fs1007insC, R702W, G908R, N289S) in *NOD2*, a *CARD9* essential splicing variant (1434 + 1G > C) and so on.

**Table 2:** Putative causal variants with PIP > 95% for autoimmune disorders. See Table 1 for trait abbreviations

Trait	Variant	Gene	Function	PIP
CD	rs2066844	*NOD2*	R702W	99.9%
CD	rs2066845	*NOD2*	G908R	99.9%
CD	rs5743293	*NOD2*	Fs1007insC	99.9%
CD	rs61839660	*IL2RA*	Intronic	99.9%
CD	rs7307562	*LRRK2*	Intronic	99.9%
CD	rs5743271	*NOD2*	N289S	99.3%
CD	rs72796367	*NOD2*	Intronic	98.3%
CD	rs41313262	*IL23R*	V362I	97.3%
CD	rs28701841	*PRDM1*	Intergenic	97.1%
UC	rs6017342	*HNF4A*	Intergenic	99.9%
UC	rs35667974	*IFIH1*	I923V	99.4%
UC	rs4676408	*GPR35*	Intergenic	99.4%
IBD	rs6062496	*RTEL1-TNFRSF6B*	Intronic	99.6%
IBD	rs141992399	*CARD9*	1434 + 1G > C	99.5%
IBD	rs74465132	*IKZF1*	Intergenic	99.4%
IBD	rs10748781	*NKX2-3*	Intergenic	99.0%
IBD	rs35874463	*SMAD3*	I170V	98.9%
IBD	rs1887428	*JAK2*	Intergenic	97.4%
SLE	rs2736100	TERT	Intronic	100.0%
SLE	rs2431697	*PTTG1-MIR146A*	Intergenic	99.9%
SLE	rs2297550	*IKBKE*	TF binding site	99.7%
SLE	rs7097397	*WDFY4*	Arg1816Gln	99.3%
SLE	rs2205960	*TNFSF4*	Intergenic	95.7%
T1D	rs34536443	*TYK2*	P1104A	100.0%
MS	rs533259	*RNASEL*	Intronic	100.0%
MS	rs733724	*HACE1*	Intronic	98.0%
PSOR	rs17716942	*KCNH7*	Intronic	100.0%
PSOR	rs12188300	*IL12B*	Intergenic	100.0%
PSOR	rs33980500	*TRAF3IP2-AS1*	D10N	100.0%
PSOR	rs11795343	*DDX58*	Intronic	99.7%
PSOR	rs8016947	*NFKBIA*	Intergenic	100.0%
PSOR	rs28998802	*NOS2*	Intronic	100.0%
PSOR	rs34536443	*TYK2*	P1104A	99.6%%
CeD	rs1893592	*UBASH3A*	Intronic	98.0%

Coding variants play a critical role in autoimmune disorders. We have observed a clear enrichment of coding causal variants for IBD compared with synonymous variants. This observation is consistent with the allelic series observed in earlier IBD genetics studies, for example in *NOD2* and *CARD9*. Coding variants in general have larger effect sizes on diseases (e.g., fs1007insC has OR close to 3 for CD) and are particularly valuable in connecting genetic findings to their biological mechanisms [86]. Coding variants have also been fine-mapped for other autoimmune disorders revealing key mechanistic insights. For example, the *IFIH1* I923V variant was mapped as the putative causal variant for T1D and UC (though only to the single-variant resolution in UC), suggesting the antiviral response pathway could be relevant to onset of these disorders. Genes with fine-mapped coding variants, such as *NOD2* and *TYK2*, are also historically known to be responsible for Blau syndrome [87] (dominant) and immunodeficiency [88] (recessive) respectively, suggesting converging biological mechanisms between polygenic and Mendelian immune disorders.

The majority of autoimmune GWAS loci implicate the noncoding genome. Farh et al. [53] first connected these noncoding genetic variations to immune-cell enhancers and found many of them gain histone acetylation or transcribe enhancer-associated RNA upon immune stimulation. Huang et al. [89] further investigated the noncoding IBD putative causal variants and found them disrupt transcription factor binding sites, implicating epigenetic marks in specific immune cells in CD patients and in gut mucosa in UC patients. The IBD noncoding variants were also found to regulate gene expressions but only in cell types or tissues relevant to the disease, not in whole blood. Despite these initial insights, the biological and molecular mechanism for most fine-mapped causal variants is still unclear, reflecting our limited knowledge in the noncoding genome.

We note that several IBD genes have multiple independent variants associated with the disease [89]. The most notable one is *NOD2*, the first reported IBD genetic association, which hosts more than ten variants contributing to the IBD risk (mostly CD). The other notable gene is *IL23R*, hosting five independent causal variants (three coding and two noncoding) that confer protection to IBD. Such a spectrum of disease-associated alleles, or allele series, can be used to establish the function-phenotype dose–response relationship, which has been shown to be important in revealing the disease genetic mechanism and facilitates the discovery and validation of therapeutic targets [86, 90, 91].

We also note that many autoimmune disease causal variants are highly pleiotropic [89]. For example, the *TYK2* P1104A variant confers protection to CD, MS, PSOR, RA, and T1D (though only mapped to single-variant resolution for T1D and PSOR). Interestingly, one causal variant can sometimes confer different directions of effects for different autoimmune or infectious disorders. For example, the *IFIH1* I923V variant increases an individual’s risk for UC but decreases the risk for T1D; an *IL2RA* intronic variant, rs61839660, increases the disease risk for CD and SLE but confers protection to T1D; the *TYK2* P1104A variant, despite being protective for several autoimmune disorders, increases homozygous carriers’ risk to tuberculosis across diverse ancestral populations [92, 93]. These observations reflect the shared biological pathways underlying autoimmune disorders and the delicate balance between tolerance and autoimmunity in the human immune system.

## Future perspectives

We have reviewed the basis of statistical fine-mapping methods, key fine-mapping studies in autoimmune disorders, and their important findings. These studies have revealed important causal variants underlying the human autoimmune disorders, and the mechanisms through which they modify individual’s risk to the diseases. Despite these successes, we note that not every autoimmune disease genetic loci have been fine-mapped and not all resources available have been leveraged in fine-mapping. This is partially because a high-quality fine-mapping typically requires a sample size larger than that of GWAS, and genetic data of higher quality to allow every variant to be assessed for their causality (while GWAS is typically tolerant to missing a few variants). Future investigations into how to properly perform fine-mapping across studies with different design factors (e.g., xpop) or genomic technology (various arrays, whole exome, or genome sequencing), as discussed in the “Further extension of statistical fine-mapping methods,” is key for fine-mapping studies to be more inclusive and powerful.

We noted that although the causal variant can be identified without ambiguity from statistical fine-mapping, they often have no clearly known functional implications if located in the noncoding genome, especially when functional priors are not incorporated. Expanding regulatory genome resources across diverse human cell types [94, 95] to advance our knowledge in the noncoding genome and incorporating those into fine-mapping frameworks are necessary to translate the putative causal variants from fine-mapping into mechanistic insights.

Lastly, MHC is a locus of paramount importance to autoimmune disorders [96, 97] but often excluded in recent statistical fine-mapping studies. This is because the MHC locus is very complex: with linkage-disequilibrium over megabses of genomes, and with complicated structural and copy number variations not often observed in other parts of the genome [98]. Thus, fine-mapping using arrays or shotgun sequencing technologies tends to be less productive. A strategy imputing the HLA alleles using data from the high density genotyping array and a set of reference individuals with HLA alleles has been shown to be productive for RA [99] and IBD [100].

Overall, fine-mapping studies for autoimmune disorders have been very productive. They have pinpointed disease causal variants and revealed key insights into the disorders. Building on this success, developments in fine-mapping methods to incorporate studies with various design factors, and resources to interpret the functional impact of causal variants on the molecular and physiological levels, will likely further advance fine-mapping studies and facilitate the therapeutics translation of their findings.

## Appendix

**Box. 1** Overview of GWAS and statistical fine-mapping models.

(We are not including intercept and covariates term in the below equations, for simplicity).

In GWAS, we test one variant at a time for its association with the phenotype of interest:

$${\varvec{y}} =\beta \cdot {\varvec{x}} + \epsilon$$

where ${\varvec{y}}$ = is the $n\times 1$ phenotype vector corresponding to $n$ individuals, $x$ is the $n\times 1$ vector denoting the genotype dosages of $n$ individuals at a specific variant position (for each individual, 1 if the individual carries the alternate allele of the variant in heterozygote, 2 if homozygote, and 0 otherwise), $\beta$ is the effect size of the variant (scalar), and $\epsilon$ is a noise term vector (typically normally distributed) of size $n\times 1$.

In contrast, when we perform statistical fine-mapping, we consider a set of $m$ variants in a locus at a time:

$$y ={\varvec{X}}\cdot{\varvec{\beta}}+ \epsilon$$

where $X$ is a matrix of size $n\times m$, and $\beta$ is now a vector of size $m\times 1$.

Not all the variants in a locus are likely causal. We typically assume sparse causal configuration, which means most of the elements of $\beta$ are zero:

$$\beta =\gamma \cdot b$$

where $\gamma$ is the causal indicator vector (1 if a variant is causal, 0 otherwise) with most of the elements being 0, and $b$ is the (true) effect sizes vector when the variant is causal for the phenotype.

In a typical Bayesian statistical fine-mapping, we set a prior distribution for the parameters $\gamma$ and $b$ such that all the elements of $\gamma$ have an equal probability of being non-zero, and each element of $b$ follows a normal distribution with pre-specified mean (= 0) and variance, to evaluate different sparse causal configurations ($\gamma$ s). In contrast, functionally informed fine-mapping corresponds to letting the prior distribution of $\gamma$ to be non-uniform depending on the variant annotations.

**Box. 2** Bayesian method overview.

(In this box, we are assuming uniform prior).

Let $X$ be the genotypes in a locus of interest (and the phenotypes), and ${X}_{i}$ be the genotype of the variant $i$ in a locus, Maller et al. [39] showed that, the Bayes factor corresponding to a model that variant $i$ is the only causal variant in the locus of interest (${M}_{i}$) over the model that no variant in the locus is causal (${M}_{0}$) depends only on the genotype data of the variant $i$:

$$B{F}_{i} = \frac{P(X|{M}_{i})}{P(X|{M}_{0})}= \frac{P({X}_{i}|{M}_{i})}{P({X}_{i}|{M}_{0})}$$

and if we assume there is exactly one causal variant in a locus of interest (i.e., the model *M* = ${M}_{1}\cup {M}_{2}\cup ...\cup {M}_{i}\cup ...$), the posterior probability of that variant being causal is simply proportional to the Bayes factor:

$$P({M}_{i}| X, M) \propto B{F}_{i}$$

Wakefield (2007,2009)^37,38^ showed that the Bayes factor can be approximated using summary statistics of the variant $i$ alone as:

$$AB{F}_{i} = \frac{P({\widehat{\theta }}_{i}|{M}_{i})}{P({\widehat{\theta }}_{i}|{M}_{0})} = \sqrt{1-{r}_{i}}\cdot exp(\frac{{{Z}_{i}}^{2}{r}_{i}}{2})$$

where $\widehat{{\theta }_{i}}$ is the marginal effect size, ${Z}_{i}$ is the z-score, and ${r}_{i}$ is the ratio of the prior variance ($W$) to the total variance of the effect size of variant $i$ ($W+{V}_{i}$) in GWAS (we have flipped the denominator and the numerator compared to the original notation of ABF in Wakefield (2007,2009), for convenience). Then the posterior inclusion probability (PIP) can be simply given as:

$$PI{P}_{i}=\frac{AB{F}_{i}}{{\sum }_{j=1}^{k}AB{F}_{j}}$$

for a locus harboring $k$ variants.

For > 1 causal variants, we cannot use such simple approximations. Let $\Gamma$ be all possible causal configurations, and ${\Gamma }_{i}$ be a subset that includes variant $i$ in the causal variant set,

$PI{P}_{i}=\frac{{\sum }_{{C\in {\Gamma }_{i}}}B{F}_{C}\cdot P(C)}{{\sum }_{{C}_{\in \Gamma }}B{F}_{C}\cdot P(C)}$. Calculating the Bayes factor for a causal configuration $C$

$B{F}_{C} = \frac{P(X|{M}_{C})}{P(X|{M}_{0})}$ over all the ${2}^{k}$ possible causal configurations as required in the calculation of denominator could be computationally expensive, and different fine-mapping methods have been developed to overcome the computational challenge (e.g., many methods restrict the number of causal variants in the model. FINEMAP^54^ performs stochastic search to avoid considering all the possible causal configurations).
